# A Randomized Controlled Trial of a Play-Based, Peer-Mediated Pragmatic Language Intervention for Children With Autism

**DOI:** 10.3389/fpsyg.2019.01960

**Published:** 2019-08-27

**Authors:** Lauren Parsons, Reinie Cordier, Natalie Munro, Annette Joosten

**Affiliations:** ^1^School of Occupational Therapy, Social Work and Speech Pathology, Curtin University, Bentley, WA, Australia; ^2^Department of Special Needs Education, University of Oslo, Oslo, Norway; ^3^Faculty of Health Sciences, The University of Sydney, Lidcombe, NSW, Australia; ^4^School of Allied Health, Australian Catholic University, Fitzroy, VIC, Australia

**Keywords:** social communication, video-modeling, intervention development, school-age, autism (ASD)

## Abstract

**Purpose:**

This randomized controlled trial evaluated the effectiveness of a play-based pragmatic language intervention for children with autism.

**Methods:**

A sample of 71 children with autism were randomized to an intervention-first group (*n* = 28 analyzed) or waitlist-first (*n* = 34 analyzed) group. Children attended 10, weekly clinic play-sessions with a typically developing peer, and parents mediated practice components at home. The Pragmatics Observational Measure (POM-2) and the Social Emotional Evaluation (SEE) evaluated pragmatics before, after and 3-months following the intervention.

**Results:**

POM-2 gains were greatest for intervention-first participants (*p* = 0.031, *d* = 0.57). Treatment effects were maintained at 3-month follow-up (*p* < 0.001–0.05, *d* = 0.49–0.64). POM-2 scores were not significantly different in the clinic and home settings at follow-up.

**Conclusion:**

Findings support the combination of play, peer-mediation, video-feedback and parent training to enhance pragmatic language in children with autism.

## Introduction

The construct of pragmatic language is complex, and a consensus definition has not been established in the literature. Early theoretical work describes pragmatics as the use of language appropriate to the social context (Prutting and Kirchner, 1987); however, more recent conceptual work recognizes an interconnection between pragmatics, social cognition and emotional understanding (Fujiki et al., 2002; Adams et al., 2005; Rodas et al., 2017). For example, social cognition has been associated with conversation skills, but the nature of the relationship between the two constructs is unknown (Matthews et al., 2018). Difficulties in the language domain of pragmatics have also been significantly associated with emotional difficulties and problems with peer relations; an association that is not apparent in other domains of language (St Clair et al., 2011).

This study utilized a contemporary description of pragmatic language, defining it as behavior encompassing the social, emotional, and communicative aspects of social language (Adams et al., 2005). This definition has been operationalized in the Pragmatics Observational Measure (POM); an observational assessment of pragmatic language behaviors recognizable in children aged 5–11 years during peer-peer play (Cordier et al., 2014). Verbal and non-verbal communicative behaviors encompassed in traditional descriptions of pragmatics are operationalized within the POM (e.g., conversation initiation, topic maintenance and change, contingency, conversation repair, facial expressions, gestures, body postures, and adapting language appropriate to the context). In addition, the interconnection between communication and social and emotional understanding is recognized through the inclusion of communication behaviors related to perspective taking, recognizing and responding to the emotions of another, regulating and expressing one’s own emotions appropriately, engagement in an interaction, and cooperation to create a mutually beneficial social exchange (Cordier et al., 2014, 2019).

Impaired pragmatic language is a core feature of autism (American Psychiatric Association, 2013) and just as the construct of pragmatic language is multifaceted, so are the presenting pragmatic language impairments in the communication profile of autism. Compared with typically developing children, children with autism initiate communication and use non-verbal cues with less frequency (Mundy et al., 1986; Adams et al., 2012). Conversational problems are also reported, such as reduced reciprocity, less varied communicative acts, diminished contingency in responses to what was previously spoken, and difficulties judging the appropriate amount of language to use in conversational responses (Paul et al., 2009). Difficulty expressing emotions, taking another’s perspective during conversation, and recognizing and responding to the emotional state of others are also recounted (Begeer et al., 2008; Paul et al., 2009).

Pragmatic language behaviors, per the definition adopted by this study, are associated with crucial friendship qualities in childhood. Cooperation, intimacy and trust distinguish friends from non-friends during childhood (Gifford-Smith and Brownell, 2003) and social conversation, verbal and non-verbal expressions of emotions, and cooperative skills are described as behavioral markers related to these characteristics of friendships (Bauminger et al., 2008). Children with autism have reported feelings of loneliness and poorer quality friendships than their typically developing peers (Bauminger and Kasari, 2000), thus facilitating quality social interactions between children with autism and peers through a focus on pragmatics might encourage the development of quality friendships that serve to promote a sense of self-worth and resilience in childhood (Gifford-Smith and Brownell, 2003). The impact of pragmatic language difficulties on social participation continues through the lifespan for individuals with autism (Tobin et al., 2014). It is therefore imperative that interventions are available to target pragmatic language at all stages of development. The complexity of an individual’s social environment increases with age, placing greater demands on an individual’s social interaction skills at each developmental stage. The focus of this study is a new pragmatic language intervention for school-aged children with autism (ages 6–11 years) as there is a paucity of intervention research targeting pragmatics in older children.

A recent systematic review and meta-analysis of pragmatic language interventions for children with autism identified 10 interventions targeting this age group (Parsons et al., 2017). The review found that most current interventions for older children target a narrow range of the pragmatic language skills included in contemporary definitions of the construct. Eight of the 10 interventions for older children targeted verbal and non-verbal communication behaviors (e.g., conversation initiation, facial expressions). Just two interventions included commination behaviors related to social-emotional skills, an important element of the evolving understanding of pragmatics.

Intervention techniques included in existing interventions for school-aged children with autism are varied. Computer based training exercises are becoming a popular approach for targeting emotion recognition skills through non-verbal cues, with mixed findings of effectiveness (Beaumont and Sofronoff, 2008; Hopkins et al., 2011; Thomeer et al., 2015). Other approaches combine didactic instruction with structured activities for reinforcement, such as role play or workbook activities (Lopata et al., 2010, 2016; Ryan and Charragain, 2010; Soorya et al., 2015). In a novel approach, Corbett et al. (2015) trained typically developing peer actors to mediate a 10-week theater-based intervention targeting directed verbal communication, non-verbal communication, and empathic responding. DeRosier et al. (2011) evaluated a group-based social skills training program that included some parent attendance, with modules targeting conversation skills in combination with perspective taking. Social communication improvements were significant for both studies, as measured by a parent-report outcome measure (DeRosier et al., 2011; Corbett et al., 2015).

Distinctly absent from current approaches to pragmatic language interventions for school-aged children with autism is a focus on using pragmatic language during ecologically valid social interactions. Likewise, longer-term maintenance and generalization of treatment effects are under evaluated in current research (Parsons et al., 2017). The instructional techniques and practice components of current interventions have a strong focus on improving discrete aspects of pragmatic knowledge (*capacity*). Pragmatics as a language domain is context dependent, therefore it is important that interventions at all stages of development also focus on contextualizing those skills for children within important social interactions in their daily lives (*performance*).

The distinction between capacity and performance is important for this study. The International Classification for Functioning, Disability and Health (ICF; World Health Organization, 2001) provides a framework for language assessment and intervention that goes beyond considering isolated skills (capacity), to include functional outcomes for daily participation (performance) in life situations (Westby and Washington, 2017). When applied to the language domain of pragmatics, the ICF indicates that assessment and intervention should focus on both pragmatic knowledge *and* how pragmatic skills are performed in functional social contexts. The importance of assessing and targeting pragmatic performance during intervention is further emphasized by recent findings that report a discord between meta-pragmatic knowledge and pragmatic performance in some children with pragmatic language impairments (Lockton et al., 2016).

One approach to facilitating children’s learning and practice of pragmatic language is via child-led, free-play interactions with a typically developing peer. A recently developed play-based, peer-mediated intervention facilitates children’s learning and practice of pragmatics in child-led, free-play interactions with a typically developing peer. The intervention is based on a theoretical framework that models how behaviors, symptomatic in children with neurodevelopmental disorders, reduce specific elements of a child’s playfulness, and that reductions in elements of playfulness can be offset by intervention techniques that enable those elements (Cordier et al., 2009). In this approach, play is defined as an interaction between an individual and the environment (physical and social) that includes four elements: internal control, intrinsic motivation; freedom from the constraints of reality, and framing (the giving and receiving of social cues; Bundy, 2004). Informed by this model, the pragmatic language difficulties associated with autism will therefore reduce children’s playfulness by impacting the play element of framing. The techniques included in the intervention therefore are designed to address pragmatic language difficulties by enabling the play element of framing.

Techniques utilized in the intervention to enable pragmatics are self- modeling through video-feedback and -feedforward, and peer- and therapist-modeling, during child-led play activities. These intervention elements have been associated with improvements in multiple social skills domains. For example, the use of video-feedback and peer-mediation have both been associated with improvements in social communication, and skill maintenance and generalization (Bellini and Akullian, 2007; Watkins et al., 2015; Chang and Locke, 2016). The combined techniques used in the current study was first evaluated by Wilkes-Gillan et al. (2016) in an RCT evaluating the intervention for children with ADHD. Children with ADHD made significant gains in playfulness, particularly in behaviors related to empathy. Benefits were also maintained and generalized to the children’s home environment (Wilkes-Gillan et al., 2016). These improvements in emotional understanding suggest that the intervention may also be effective for targeting pragmatic language.

A systematic approach should be taken to designing and evaluating complex interventions; combining theory development, trials of feasibility, and exploratory studies that culminate in evaluations of effectiveness (Craig et al., 2008). The aforementioned intervention was found to significantly improve play skills in children with ADHD, with gains maintained at 2-month follow-up (Wilkes-Gillan et al., 2016). Recently, pilot studies have established the feasibility and appropriateness of an adapted version of this play-based intervention tailored to the needs of children with autism (Kent et al., 2018; Parsons et al., 2018). Preliminary effectiveness in the areas of pragmatic language performance and capacity were evaluated using the POM and the Social Emotional Evaluation (SEE; Wiig, 2008), respectively (Parsons et al., 2018).

This randomized controlled trial (RCT) aimed to evaluate the effectiveness of the intervention for improving pragmatic language performance and capacity in children with autism during social play with peers. Specific research questions were:

1 Do children with autism who receive a play-based, peer-mediated intervention make greater gains in pragmatic language performance (POM-2) and capacity (SEE) than children with autism who have not received a pragmatic language intervention?
2 Are changes in pragmatic language performance (POM-2) and capacity (SEE) maintained 3-months after the intervention period?
3 Is pragmatic language performance (POM-2) in play-based interactions equivalent in the clinic and home environments following the intervention?
4 Which variables moderate pragmatic language performance (POM-2) and capacity (SEE) over the duration of the study?

## Materials and Methods

### Trial Design and Registration

This RCT used two parallel groups, comprising part of a larger project also evaluating the intervention’s impact on children’s play skills. The reporting of this study was guided by the Consolidated Standards of Reporting Trials (CONSORT) guidelines (Schulz et al., 2010) to ensure transparent reporting of methodology. The Template for Intervention Description and Replication (TIDieR) guidelines (Hoffmann et al., 2014) were also considered to allow for easier intervention replication and utilization.

The trial was registered with the Australia New Zealand Clinical Trials Registry *a priori* (ACTRN12615000008527) and the protocol was approved by Curtin University’s Human Research Ethics Committee (HR04/2015). Researchers explained the study requirements to all children and parents prior to obtaining consent. Parents provided written consent on behalf of their children, and children provided verbal assent (ages < 7 years) and written consent (ages > 7 years). Recruitment took place between February 2016 and April 2017, and 3-month follow-ups were completed by October 2017.

### Participants

Recruitment occurred using convenience sampling. Fliers were distributed to schools and speech pathology and occupational therapy clinics and posted on online forums for speech pathologists and parents of children with autism. Study information was also disseminated to families waitlisted for a large, local autism service provider. Parents of 102 children with autism contacted researchers and were screened for eligibility via telephone; 80 children met the inclusion criteria and were able to commit to the study schedule.

**FIGURE 1:**
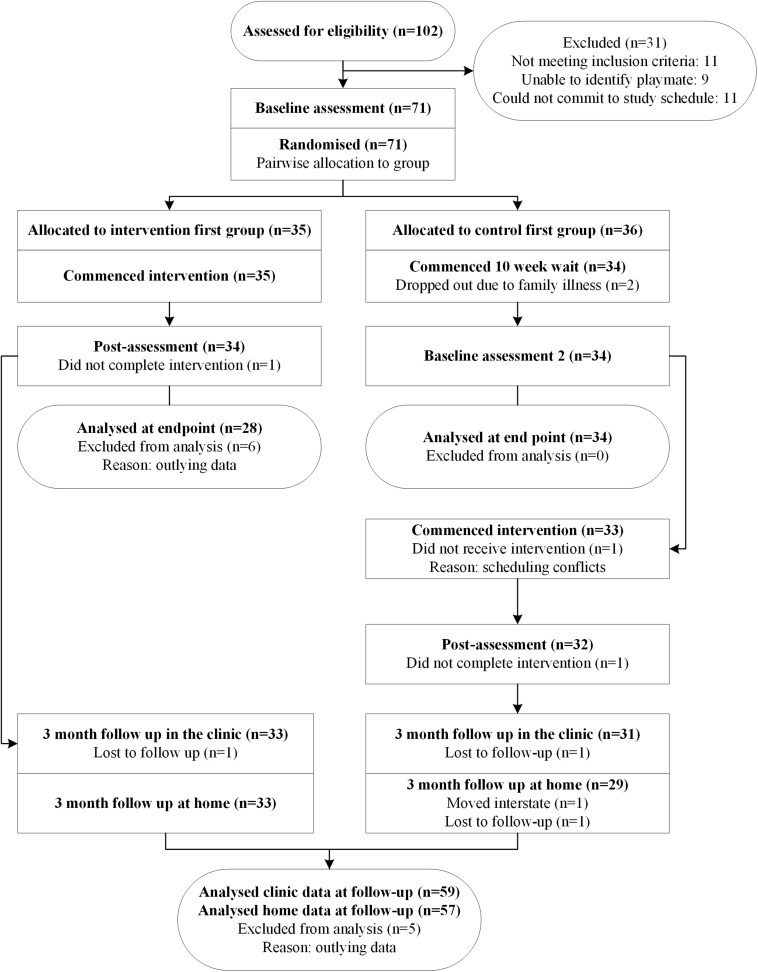
CONSORT flow chart.

To attend the study, children with autism were required to invite a typically developing playmate to attend the study. Of the 80 children screened as meeting inclusion criteria, nine were unable to identify a suitable playmate, leaving a total of 71 children who entered the study. One family enrolled three children with autism and a second family enrolled two children with autism. One intervention-first dyad (child with autism and playmate) dropped out after eight sessions and two waitlisted dyads did not return for baseline 2 due to family illness, reducing the total sample to 68 children with autism. One waitlist-first dyad did not commence the intervention due to scheduling conflicts and another dropped out after seven sessions. A total of 66 children completed the intervention. See Figure 1 for the participant flow diagram. Three typically developing playmates attended the intervention twice; each time with a different child with autism. Three playmates who dropped out were replaced with three new playmates. See Table 1 for demographic information for all children and parents.

**TABLE 1:** Participant demographic variables.

	**Children with autism**			**Playmates**		
	**Intervention-First**	**Waitlist-First**	***p***	**Intervention-First^a^**	**Waitlist-First^b^**	***p***
**Parent demographics^c^**						
Age (years)	42.4 (5.92)	40.6 (3.94)	0.170	42.5 (5.68)	39.8 (6.67)	0.108
Education after high school	20 of 26	26 of 33	0.906	20 of 26	29 of 35	0.877
**Child demographics**						
Age (years)	8.6 (1.38)	8.4 (1.36)	0.558	8.6 (1.83)	8.0 (1.48)	0.185
Gender (male)	26 of 28	28 of 34	0.220	12 of 27	20 of 35	0.321
**Screening assessments**						
*CCBRS*^d^						
Autistic disorder	86.0 (7.88)	85.5 (7.37)	0.819	50.5 (10.12)	55.7 (15.92)	0.179
Asperger’s disorder	81.1 (11.00)	79.0 (11.06)	0.492	50.2 (9.39)	53.1 (12.16)	0.350
ADHD (inattentive)	75.5 (11.42)	81.5 (8.97)	0.037^∗^	57.2 (16.43)	59.0 (13.91)	0.668
ADHD (hyperactive-impulsive)	74.8 (13.70)	69.9 (15.25)	0.217	54.6 (13.17)	57.5 (15.43)	0.460
*CCC-2^e^*						
General Communication Composite	40.3 (11.62)	35.4 (17.15)	0.248	74.1 (20.02)	73.0 (21.69)	0.932
Social Interaction Difference Index	−11.4 (9.18)	−4.4 (8.02)	0.006^∗∗^	0.3 (7.70)	0.5 (7.41)	0.363
*EVT-2*	104.8 (13.16)	104.4 (12.50)	0.908	107.8 (11.87)	110.3 (11.56)	0.432
*TACL-4*	8.6 (2.42)	8.4 (2.09)	0.844	8.7 (1.47)	9.2 (1.95)	0.306
**Dyad variables**						
Age difference (months)	1.68 (23.4)	−5.8 (19.44)	0.177			
Playmate sibling (%)	19 of 28 (67.9)	28 of 34 (82.4)	0.233			

*CCBRS, Conners Comprehensive Behavior Rating Scale; CCC-2, Children’s Communication Checklist 2nd Edition; EVT-2, Expressive Vocabulary Test 2nd Edition; TACL-4, Test for Auditory Comprehension of Language 4th Edition; ^*a*^Number of playmates not equal to number of children with autism as two playmates attended twice with different children with autism, and one dropped out and was replaced by a new playmate; ^*b*^Number of playmates not equal to number of children with autism as one playmate attended with two different children with autism, and two dropped out and were replaced by two new playmates; ^*c*^Some parents enrolled multiple children with autism, and one parent had two children enrolled as playmates; ^*d*^Clinical cut off = T-score > 70, borderline clinical cut off = T-score > 65; ^*e*^General Communication Composite < 55 and a Social Interaction Difference Index < 0 suggests a communication profile indicative of autism; ^∗^*p* < 0.05; ^∗∗^*p* < 0.01.*

#### Children With Autism

Children with autism needed to be aged 6–11 years to participate and have a diagnosis of autism or Asperger syndrome in accordance with the DSM-IV or 5 (American Psychiatric Association, 2000, 2013) without an intellectual disability. To receive an autism diagnosis in Western Australia, children are assessed by a psychiatrist or pediatrician, psychologist and speech pathologist who then collaborate to make a joint diagnostic decision that the child meets the DSM diagnostic criteria (Glasson et al., 2008). Researchers sighted these multidisciplinary diagnostic reports to confirm children’s autism diagnoses and absence of an intellectual disability. As severe structural language difficulties may reduce children’s comprehension of intervention concepts, a standard score ≥ 70 on the Expressive Vocabulary Test (EVT-2; Williams, 2007) and scaled score ≥ 4 on the Elaborated Sentences and Phrases subtest of the Test for Auditory Comprehension of Language (TACL-4; Carrow-Woolfolk, 2014) were additional eligibility requirements. Parents of children with autism identified improving social communication and play skills as goals for their children.

#### Playmates

Children with autism invited a typically developing peer to attend the trial as a playmate. Informed by pilot studies (Henning et al., 2016; Kent et al., 2018; Parsons et al., 2018), peers needed to be known to the child with autism (i.e., sibling or friend), and of a similar age; ideally within 2 years. A majority (75.8%) of playmates in the study were siblings of the children with autism. The remainder were friends with the exception of three cousins. Playmates were required to be aged 6–11 years, with no parental concern for neurodevelopmental disorders. An EVT-2 standard score ≥ 70, and a TACL-4 Elaborated Sentences and Phrases scaled score ≥ 4 were also required to ensure playmates did not have severe structural language difficulties that might reduce comprehension of intervention concepts.

### Instruments

#### Screening Measures

The *Expressive Vocabulary Test, 2nd Edition* (EVT-2; Williams, 2007) and the *Elaborated Phrases and Sentences* subtest of the *Test for Auditory Comprehension of Language 4th Edition* (TACL-4; Carrow-Woolfolk, 2014) were used to screen children’s structural language. The EVT-2 is a measure of word recall and expressive vocabulary with strong internal consistency (α = 0.96), and test–retest reliability (*r* = 0.95). EVT-2 standard scores show moderate to strong correlations with Clinical Evaluation of Language Fundamentals, 4th edition (Semel et al., 2003) standard scores (*r* = 0.68–0.80; Williams, 2007).

*Elaborated Phrases and Sentences* evaluates receptive syntax. The TACL-4 has sensitivity and specificity indices of 0.22 and 1.00, respectively, for detecting children with language impairment at the selected cut-off.

Two parent report measures were used to characterize the communication and behavior profiles of the children with autism and to confirm there were no developmental concerns for the playmates. The *Children’s Communication Checklist 2nd Edition* (CCC-2; Bishop, 2006) evaluated language form, pragmatics, and semantics, and the *Conners Comprehensive Behavior Rating Scales* (CCBRS; Conners, 2008) assessed behavioral, emotional, academic and social problems in children and adolescents. The CCC-2 has sensitivity and specificity values of 0.89 and 0.97, respectively, for identifying children with autism symptomology and pragmatic language impairment (Bishop, 2006). The CCBRS has good evidence for internal consistency (α = 0.67–0.97), test–retest reliability (*r* = 0.56–0.96), and inter-rater reliability (*r* = 0.50–0.89), and overall correct classification rates of 0.70–0.89 for its clinical indexes (Conners, 2008).

#### Performance Outcome Measure

The *Pragmatics Observational Measure 2* (POM-2; Cordier et al., 2014, 2019), was the primary outcome measure. It is an observational instrument that evaluates pragmatic language performance during social play and can be used by blinded assessors to reduce measurement bias. Items are rated on a four-point scale; higher scores indicate more advanced pragmatic language competence. In this updated version of the POM, an Overall Measure score and two subscale scores (Non-verbal Communication and Verbal Communication) are produced. The POM and POM-2 have strong evidence for internal consistency (α = 0.99), and construct validity (99% of items and 97% of people fit Rasch expectations) (Cordier et al., 2014, 2019). Criterion validity was assessed against the Pragmatic Protocol (Prutting and Kirchner, 1987), and was found to be strong (*r* = 0.95, *p* = 0.005; Cordier et al., 2014). The Pragmatic Protocol was the only psychometrically validated observational measure of pragmatic language at the time the POM was validated.

To evaluate the pragmatic language performance of children with autism and their playmate, 15-min videos of each dyad playing in the clinic playroom were recorded pre and post intervention, and at 3-month follow up. Waitlist-first dyads were also filmed playing in the clinic 10-weeks prior to starting the intervention. Additional play footage was recorded at the homes of children with autism at 3-month follow-up to compare performance across environments. De-identified videos were sent to an independent assessor for rating. The assessor was blinded to study purpose, group allocation, participant diagnosis, and timing of the videos. Rasch analysis determined the assessor’s scores were reliable for the 310 videos sampled, as goodness-of-fit statistics were within the required parameters (*MnSq* < 1.4 and > 0.7; standardized value < 2.0).

#### Capacity Outcome Measure

The *Social Emotional Evaluation* (SEE; Wiig, 2008) measured the pragmatic language capacity of the children with autism and their playmates. It is criterion-referenced, providing *z*-scores for ages 6; 0–7; 11, 8; 0–9;11, and 10; 0–12; 11. The four core subtests were administered; each containing receptive and expressive tasks: *Identifying Common Emotions*, *Recognizing Emotional Reactions*, *Understanding Social Gaffes*, and *Understanding Conflicting Messages.* Subtest raw scores are summed and converted to *z*-scores producing receptive, expressive and total composite scores. The SEE has demonstrated good internal consistency (α = 0.76–0.88), test–retest reliability (*r* = 0.88–0.93), and inter–rater reliability (*r* = 0.96–1.00; Wiig, 2008). At a *z*-score cut-off of −1.00 the SEE has overall sensitivity and specificity values of 0.95–1.00, for identifying children with autism.

### Procedures

The necessary sample size for this study was calculated using G^∗^Power (Faul et al., 2007). A sample size of 34 participants per group was needed to detect a moderate-to-large effect size (Cohen’s *d* ≥ 0.7) with 80% power using a *t*-test with an alpha of 0.05 (two tailed significance).

#### Randomization

Participants were randomized in pairs, as recruitment was sporadic. An independent researcher used a random number generator (random.org; Haahr, 2010) to allocate participants to group 1 (intervention-first) or group 2 (waitlist-first). Group allocation was concealed in envelopes until baseline assessments were completed to ensure researchers, participants and assessors were blinded to group allocation at baseline. Intervention-first participants attended the intervention immediately (*n* = 35). Waitlist-first participants waited for 10-weeks before starting the intervention (*n* = 34). All participants agreed not to undertake any pragmatics and play interventions while participating in this study. To avoid contamination between groups, families received the same group allocation if they enrolled multiple children with autism at the same time (*n* = 4). This was also done to avoid burdening families with an extended intervention period if children were allocated to different groups.

#### Baseline Assessment

Week one of participation included the following baseline assessment procedures. Dyads entered the clinic playroom to play for 15-min. The play session was filmed to allow for a blinded assessor to rate both children’s pragmatic language performance using the POM-2. The playroom contained a variety of toys and equipment to encourage social-play activities such as role playing, board games, construction, or gross-motor play. A list of available toys is reported in Parsons et al. (2018). Therapists and parents observed dyads playing via a computer screen in an adjacent room, and the therapist consulted with parents about their child’s social communication difficulties. Following the play, children with autism and their playmates completed the EVT-2, TACL-4 and SEE, and parents were provided with parent-report questionnaires (i.e., CCC-2 and CCBRS).

#### Intervention: Clinic Components

Dyads attended weekly intervention sessions with a therapist at Curtin University. Additional appointments were scheduled for children who missed sessions to optimize participation. A speech pathologist and an occupational therapist conducted the eight intervention sessions between pre- and post- assessment (sessions 1 and 10, respectively). Both therapists received training in the intervention during the pilot study with 10 participants and were supported by the second author. Children were allocated to a therapist based on mutual availability. Of the children who completed post- assessments (*n* = 66), 97% attended eight intervention sessions. Two participants had post- assessments after six intervention sessions, and one after seven sessions, as the families were unable to commit to additional weekly appointments. On average, participants completed eight intervention sessions in 8.3 weeks.

All weekly clinic sessions followed the same format: (1) 15–20 min of therapist-lead video-feedback; (2) 20 min of child-lead play with therapist modeling; and (3) 15 min of therapist-parent discussion while children continued playing. Toys in the playroom were selected to suit a range of ages, play skill levels, and interests. There were two wall-mounted video cameras fitted in the playroom to film all intervention play sessions for use in video-feedback.

During video-feedback, dyads viewed 3–4 clips of video footage (30–60 s each) from their previous week’s play session, coded as “red play” or “green play,” and discussed observed pragmatic language skills with the therapist. Parents were present during these video-feedback discussions. “Green play” exemplified pragmatic language that promoted social interaction (e.g., responding to questions, making suggestions to evolve the play, using body posture to demonstrate engagement in the interaction). The pragmatic language viewed in “red play” did not promote social interaction (e.g., rejecting playmate’s suggestions, tangential discourse, failure to consider playmate’s perspective or emotions). Therapists and children discussed the pragmatic language skills exemplified in green play, and the skills that could promote the social interaction in red play. Video-feedback ended with video-feedforward in the form of 2–3 pragmatic language skills to put into practice in the playroom that day. Therapists created the video-feedback sequences between children’s intervention sessions by editing the digital video files recorded by cameras *in situ* in the playroom using video editing software (Adobe Premier Pro CC; Adobe Systems Incorporated, 2014).

The therapists entered the playroom with the dyads following video-feedback and played with the dyad as a playmate, rather than an instructor, to ensure activities were child-led. Parents viewed the play in an adjacent room on a computer screen. While playing, the therapist ensured that activities remained as play (based on the adopted model; Bundy, 2004), but moved in a direction that promoted the intervention goals. Therapists promoted intervention goals by modeling targeted pragmatic language skills to children with autism (e.g., sharing a new play idea if conversation initiation or maintenance was a target) and strategies for supporting another’s pragmatic language to playmates (e.g., asking questions if the child with autism did not provide enough information about their play idea). After 20-min, therapists joined the parents in an adjacent room to discuss their child’s intervention goals and strategies to promote targeted pragmatic language principles at home.

Pragmatic language targets were informed by the pragmatic language behaviors operationalized by the POM-2, and individualized targets were selected by the therapists and tailored to each dyad based on POM-2 performance. A list of all possible targets is provided in Table 2. Challenges in the pragmatic language performance profile of the child with autism (based on POM-2 baseline scores) were considered in relation to their playmate’s pragmatic language performance (also based on POM-2 baseline scores). In doing so, a playmate’s pragmatic language performance could be leveraged both as a model and facilitator of performance for the child with autism. Pilot-studies indicated that children recalled principles more easily when presented as short, syntactically simple “catch phrases” (Parsons et al., 2018). Prior to commencing this RCT, researchers developed a matrix of catch phrases representing all possible target skills (e.g., “share ideas” if conversation initiations were targeted). Therapists used these phrase labels when discussing the pragmatic language principles during video-feedback.

**TABLE 2:** Pragmatic language skills targeted by the intervention studied.

**Pragmatic language skill**
Introducing communication and being responsive to a playmate’s communication: • Selecting a range of conversation topics • Conversation topic maintenance and change • Contingency with previously communicated content Initiating verbal communication • Responding to playmate’s communication • Repairing or revising communication to resolve breakdowns
Using non-verbal communication and interpreting a playmate’s non-verbal communication: • Using and responding to facial expressions • Using and responding to gestures (i.e., body movements or actions) • Using and responding to body positioning • Using physical space between playmates appropriately
Understanding and responding to the emotional reactions and intentions of a playmate: • Being aware of and responsive to playmate’s emotional needs • Integrating playmate’s perspective or emotions • Using verbal and non-verbal language appropriate to the social context • Adapting behavior and language to environmental demands
Using cognitive processes to promote an interaction with a playmate • Attending to playmate’s communicative content, planning and initiating appropriate responses • Planning and delivering organized communication content
Using negotiation techniques to promote an interaction with a playmate: • Resolving conflicts • Cooperating to promote a mutually beneficial exchange • Engagement in play-based interaction with playmate • Effectively expressing viewpoint, emotions or opinions • Making suggestions and effectively offering opinions • Disagreeing effectively so that the interaction is continued

To maintain fidelity during the intervention, therapists worked closely with each other to set intervention goals, debrief between intervention sessions, review the language used to talk to children about pragmatic language skills. Therapists also viewed each other delivering the intervention to provide feedback and discuss consistent use of techniques.

#### Intervention: Home Components

Therapists trained parents in the home-based intervention components during session 1. Parents were provided with a manual to review each week, containing ten modules on social communication and play skills that are challenging for children with social difficulties (e.g., perspective taking, negotiation and problem solving). Each module defined the focus skill, explained its importance at home and school, and described strategies for parents to use to support their child’s social play. Therapists prescribed one module to parents each week based on observed challenges in the playroom and problems occurring at home or school.

Families were also given a series of short videos (6–8 min) aligned with the modules contained within the manual. Parents and children with autism viewed one video per week at home. The videos portrayed the play-based interactions of two fictional characters in contexts familiar to children (e.g., playground, park, at home). The videos included examples of red and green play and the characters received help from superheroes to resolve red play before modeling how to repair the social interaction. Parents guided a discussion with their child about the play and social communication skills and strategies observed. Information about the manual and videos will be made available by the authors upon request. Parents were instructed to arrange weekly playdates for dyads between clinic sessions.

Through discussions with the therapist and the parent manual, parents were coached to provide feedback before, during and after the playdate using the language and terminology that the therapist used during clinic sessions. Through weekly discussion with parents, it was clear that parents were highly compliant with reading the prescribed chapters, viewing the videos with their child and following through on arranging playdates for their children, however, compliance was not formally assessed.

#### Post-intervention and Follow-Up Assessment

Participation week 10 included post-intervention assessments (i.e., POM-2 and SEE), conducted mirroring baseline procedures in the clinic. The same procedures were completed at the clinic 3-months later. Therapists also attended the homes of children with autism in the week proceeding their clinic follow-up, to film dyads playing for 15-min using hand-held cameras. This allowed for the blinded assessor to rate children’s pragmatic language performance (POM-2) in a secondary environment at follow-up. Play at home included outdoor or indoor play with the children’s own toys.

### Statistical Analysis

#### Data Preparation

Ordinal POM-2 item ratings were converted to interval level measure scores using Rasch analysis in Winsteps (Version 3.92.0; Linacre, 2016). Measure scores for POM-2 Overall, and the Non-verbal and Verbal Communication subscales were derived for each participant for all assessment time-points. POM-2 and SEE scores of participants with TACL-4 scores of 4 (i.e., at inclusion cut-off; *n* = 7) and participants who attended < 10 sessions prior to post- assessment (*n* = 2) were reviewed. Person-fit statistics did not fit Rasch expectations for all POM-2 measure scores at all time points for four participants and so they were excluded from analysis as their data was not considered reliable. SEE composite *z*-scores were below floor level for a further two participants, so they were excluded from analysis as a true baseline could not be established. The remaining analyses of participant demographic, screening and outcome measure data were performed using IBM SPSS Statistics (Version 22; IBM Corporation, 2013).

#### Baseline Differences

Shaprio–Wilkes tests indicated data were normally distributed, so independent samples *t*-tests for interval level variables or Pearson’s Chi Square tests for categorical variables were used to compare baseline demographic and screening data of children in each group. Parent and playmate data were equivalent between groups. The demographic, language and behavioral profiles of children with autism did not differ, with the exception of their Inattentive ADHD and Social Interaction Deviance Composite (SIDC) scores. While the group means for these two scores differed, the scores of both groups fell within the same clinical categories defined by the cutoff scores of each measure. The Inattentive ADHD *T*-scores for both groups were above the clinical cut-off score of 70. The SIDC for both groups was < 0, which in combination with a General Communication Composite < 55 suggests a communication profile characteristic of autism (see Table 1).

#### Differences in Change Between Groups

Change-scores was calculated for POM-2 Overall, POM-2 Non-verbal Communication, POM-2 Verbal Communication, SEE Receptive, SEE Expressive and SEE Total scores by deducting baseline from post scores (for intervention-first participants; *n* = 28) or baseline one from baseline two scores (for waitlist-first participants; *n* = 34). Independent samples *t*-tests compared the difference in the change-score means of both groups. Significance was set at *p* < 0.05. Cohen’s *d* effect sizes were calculated, and interpreted as follows: 0.2 = small effect size, 0.5 = medium effect size, 0.8 = large effect size (Cohen, 1988).

#### Changes Over Time

To increase the statistical power of the remaining analyses, pre, post and 3-month follow up POM-2 and SEE scores for all participants (*n* = 59) were combined. Linear mixed models were created for each score (i.e., POM-2 Overall, POM-2 Non-verbal Communication, POM-2 Verbal Communication, SEE Receptive, SEE Expressive and SEE Total) to assess the fixed effect of time, allowing for subject level random intercepts. Pairwise comparisons of main effects between each time point were assessed if a significant overall main effect of time was detected. Significance was set at *p* < 0.05. Cohen’s *d* effect sizes were calculated and interpreted using the previously described convention.

#### Pragmatic Language Performance Across Environments

A difference-score was calculated for all POM-2 scores (Overall, Verbal and Non-verbal) at 3-month follow-up by deducting home follow-up scores from clinic follow-up scores. Single sample *t*-tests were conducted on the difference-scores to determine whether they were significantly different from zero. Pragmatic language performance during play-based interactions with a peer was considered to be equivalent across environments at the end of the study if results were not significant (*p* > 0.05).

#### Moderator Analysis

An exploratory moderator analysis was conducted using linear mixed models. Six potential moderating variables were examined: time (i.e., pre, post, and follow-up), expressive vocabulary (EVT-2 score), receptive syntax (TACL-4 score), playmate relationship (sibling, non-sibling), age difference between children within the dyads, age group of children with autism (i.e., 6–7, 8–9, 10–11 years; age categories mirrored those used in the SEE *z*-scores), and therapist profession (speech pathologist, occupational therapist). These variables were selected as they represent child, dyad and therapist characteristics that might influence children’s pragmatic capacity and performance during the intervention. Dependent variables examined were POM-2 Overall, POM-2 Non-verbal Communication, POM-2 Verbal Communication, SEE Receptive, SEE Expressive and SEE Total scores, allowing for subject level random intercepts. Time was the independent variable.

As there was no *a priori* hypothesis for entering variables into the model, univariate models first assessed the significance of each moderating variable as a means of screening for moderators to include in the final multivariate analysis. Then, significant univariate variables were entered into a multivariate model. As there was no *a priori* hypothesis for entering variables into the model, non-significant independent variables were removed from the model until only significant explanatory variables remained. Significance was set at *p* < 0.05.

## Results

### Differences in Change Between Groups

The overall pragmatic performance change in children with autism in the intervention-first group over the 10-weeks of intervention was significantly greater than the change in the waitlist-first group during their 10-week waiting period, *t*(60) = 2.213, *p* = 0.031, *d* = 0.57. Changes in non-verbal communication were also significantly greater for the intervention-first group compared to the waitlist-first group over the same time period, *t*(60) = 2.676, *p* = 0.010, *d* = 0.68. A small to medium effect was detected in favor of the intervention-first group when comparing changes-scores for Verbal Communication, SEE Receptive, SEE Expressive and SEE Total composites; however, between-groups differences were not significant. Full results are presented in Table 3 and the Supplementary Figure S1.

**TABLE 3:** Comparison of intervention-first group change scores with waitlist-first group change scores.

**Measure**	**Intervention-First *Mean (SD)***		**Waitlist-first *Mean (SD)***		**Change score comparisons**		**Effect size**
	**Baseline 1**	**Post-intervention**	**Baseline 1**	**Baseline 2**	***t***	***p***	***d***
**POM-2**							
Overall	26.7 (30.42)	43.6 (26.04)	16.6 (29.62)	20.7 (28.84)	2.21	0.031^∗^	0.57
Non-verbal	28.4 (33.47)	51.3 (28.74)	19.9 (31.67)	22.4 (30.60)	2.68	0.010^∗^	0.68
Verbal	17.5 (35.62)	38.9 (33.35)	3.9 (34.41)	9.7 (35.90)	1.74	0.087	0.46
**SEE**							
Receptive	−0.59 (1.13)	−0.16 (0.92)	−0.28 (1.10)	−0.20 (0.13)	1.61	0.112	0.47
Expressive	−0.62 (1.05)	−0.25 (1.03)	−0.53 (1.03)	−0.50 (1.03)	1.61	0.114	0.40
Total	−0.63 (1.16)	−0.26 (0.99)	−0.49 (1.08)	−0.35 (1.10)	1.04	0.304	0.27

*POM-2, Pragmatics Observational Measure 2nd Edition; SEE, Social-Emotional Evaluation; *SD*, standard deviation; ^∗^*p* < 0.05; Cohen’s *d* interpretation: 0.2 = small, 0.5 = medium, 0.8 = large.*

### Change Over Time

A significant main effect of time was detected for children with autism on: (a) POM-2 Overall, *F*(2,119) = 22.381, *p* = < 0.001; (b) Non-verbal Communication, *F*(2,119) = 21.041, *p* = < 0.001, and (c) Verbal Communication scores, *F*(2,119) = 18.860, *p* = < 0.001. Pairwise comparisons showed overall pragmatic language, non-verbal communication, and verbal communication performance improved significantly pre to post intervention and pre to 3-month follow-up in the clinic, with medium effect sizes. POM-2 scores increased between post and 3-month follow-up, however, changes were not significant (Table 4). Results indicate that treatment effects for pragmatic language performance were maintained.

**TABLE 4:** Comparison of outcome measures over time.

			**Estimated marginal means**			**Pairwise comparisons^a^**					
				
	**Fixed effect of time**		**Pre-**	**Post-**	**3-month follow-up**	**Pre-post**		**Pre-follow-up**		**Post-follow-up**	
							
**Measure**	***F***	***p***	***Mean (SE)***	***Mean (SE)***	***Mean (SE)***	***p***	***d***	***p***	***d***	***p***	***d***
**POM-2**											
Overall	22.38	< 0.001^∗∗∗^	23.4 (3.73)	45.5 (3.78)	49.3 (3.80)	< 0.001^∗∗∗^	0.54	< 0.001^∗∗∗^	0.63	0.360	0.09
Non-verbal	21.04	< 0.001^∗∗∗^	25.1 (4.05)	49.3 (4.11)	53.7 (4.14)	< 0.001^∗∗∗^	0.54	< 0.001^∗∗∗^	0.64	0.354	0.10
Verbal	18.86	< 0.001^∗∗∗^	12.2 (4.63)	37.2 (4.70)	43.9 (4.74)	< 0.001^∗∗∗^	0.49	< 0.001^∗∗∗^	0.62	0.223	0.13
**SEE**											
Total	3.78	0.026^∗^	−0.46 (0.14)	−0.11 (0.14)	−0.23 (0.14)	0.008^∗∗^	0.23	0.080	0.15	0.349	−0.08
Receptive	5.00	0.008^∗∗^	−0.35 (0.17)	0.03 (0.14)	−0.10 (0.14)	0.002^∗∗^	0.22	0.040^∗^	0.15	0.310	−0.09
Expressive	4.71	0.011^∗^	−0.54 (0.14)	−0.15 (0.14)	−0.28 (0.14)	0.003^∗∗^	0.26	0.050^∗^	0.17	0.304	−0.09

*POM-2, Pragmatics Observational Measure 2nd Edition; SEE, Social-Emotional Evaluation; SE, Standard error; ^*a*^POM-2 scores from 3-month follow-up assessment in the clinic; ^∗^*p* < 0.05; ^∗∗^*p* < 0.01; ^∗∗∗^*p* < 0.001; Cohen’s *d* interpretation: 0.2 = small, 0.5 = medium, 0.8 = large.*

There was a significant main effect of time on the: (a) SEE Total, *F*(2, 117) = 3.783, *p* = 0.026; (b) SEE Receptive, *F*(2,117) = 5.000, *p* = 0.008, and (c) SEE Expressive scores, *F*(2,117) = 4.709, *p* = 0.011. Pairwise comparisons of SEE scores showed that receptive and expressive composites improved significantly pre to post and pre to 3-month follow-up. The overall composite increased significantly pre to post intervention but not pre to 3-month follow-up. Treatment effects for pragmatic capacity were maintained at 3-month follow-up as changes from post to 3-month follow-up were not statistically significant.

### Pragmatic Language Performance Across Environments

At 3-month follow-up children’s POM-2 Overall measure scores were higher in the home (mean = 50.65, *SD* = 32.36) than the clinic (mean = 49.51, *SD* = 29.99). Likewise, Non-verbal Communication scores were greater in the home (mean = 58.27, *SD* = 34.49) than the clinic (mean = 53.93, *SD* = 32.13); however, Verbal Communication scores were higher in the clinic (mean = 44.04, *SD* = 38.35) than at home (mean = 40.15, *SD* = 42.71). Single sample *t*-tests on the difference between home and clinic scores were not significant for: (a) POM-2 Overall, *t*(56) = 0.312, *p* = 0.757; (b) Non-verbal Communication, *t*(56) = 0.1.029, *p* = 0.308, and (c) Verbal Communication, *t*(56) = −0.761, *p* = 0.450; supporting the hypothesis that the differences between clinic and home POM-2 scores were equivalent to zero.

### Moderator Analysis

Univariate main effects were explored for six variables that could potentially moderate the intervention effect as measured by the POM-2 and SEE. Variables examined were time (i.e., pre, post, and follow-up), expressive vocabulary (EVT-2 score), receptive syntax (TACL-4 score), playmate relationship (sibling, non-sibling), age difference between children within the dyads, age of children with autism (i.e., 5–7; 8–9; 10–11 years), and therapist profession (speech pathologist, occupational therapist). Playmate relationship, age difference between children in each dyad, and the age group of the child with autism (6–7; 8–9; 10–11 years) did not have a significant main effect on POM-2 or SEE scores. A significant, positive main effect of TACL-4 score was detected for all outcome scores. Higher TACL-4 score predicted greater changes in: (a) POM-2 Overall, *F*(1,57) = 15.00, *p* < 0.001; (b) POM-2 Non-verbal, *F*(1,57) = 14.18, *p* < 0.001; (c) POM Verbal *F*(1,57) = 13.34, *p* < 0.001; (d) SEE Total, *F*(1,58) = 12.93, *p* = 0.001, = 0.004; (e) SEE Receptive, *F*(1,58) = 13.66, *p* = < 0.001, and (e) SEE Expressive, *F*(1,57) = 9.08, *p* = 0.004. A significant, positive main effect was present for EVT-2 score. Higher EVT-2 scores predicted greater changes in: (a) POM-2 Overall, *F*(1,56) = 4.02, *p* = 0.05; (b) POM-2 Verbal Communication, *F*(1,56) = 5.16, *p* = 0.046; (c) SEE Total, *F*(1,57) = 25.67, *p* < 0.001; (d) SEE Receptive, *F*(1,57) = 45.47 *p* < 0.001, and (e) SEE Expressive, *F*(1,56) = 19.57, *p* < 0.001. The main effect of therapist profession was significant, favoring speech pathologist, for all POM-2 scores: (a) Overall, *F*(1,58) = 12.98, *p* = 0.001; (b) Non-verbal, *F*(1,58) = 13.59, *p* < 0.001, and (c) Verbal (*F*(1,57) = 11.00, *p* = 0.002), but not the SEE scores.

Significant predictor variables from the univariate analyses were simultaneously entered into the linear mixed models for POM-2 and SEE scores to produce a final model of variables that predicted children’s pragmatic language scores across the study. Non-significant variables were removed from the multivariate analysis through backward elimination. Final models for POM-2 and SEE scores are presented in Tables 5, 6, respectively. Significant main effects of time (i.e., pre, post, 3-month follow-up), therapist profession (i.e., speech pathologist, occupational therapist) and receptive syntax (TACL-4 score) were present for all POM-2 scores. Significant main effects of time, EVT-2 and TACL-4 were present for SEE Total and SEE Receptive scores, and time and EVT-2 were significant main effects for SEE Expressive scores.

**TABLE 5:** Results of multiple linear mixed model regression for POM-2 scores.

	**POM-2 Overall**			**POM-2 Non-verbal**			**POM-2 Verbal**		
	**Parameter estimates (95%CI)**	***F***	***p***	**Parameter estimates (95%CI)**	***F***	***p***	**Parameter estimates (95%CI)**	***F***	***p***
**Time**		21.47	< 0.001^∗∗∗^		19.93	< 0.001^∗∗∗^		18.28	< 0.001^∗∗∗^
Pre	−25.8 (−34.2 to −17.4)			−28.3 (−37.8 to −18.8)			−31.7 (−42.7 to 20.2)		
Post	−4.1 (−12.5 to 4.3)			−4.7 (−14.2 to 4.9)			−7.0 (−18.0 to 3.9)		
3-month follow-up^a^	0			0			0		
**Therapist profession**		6.50	0.014^∗^		7.05	< 0.001^∗∗∗^		7.08	0.010^∗^
OT	−13.8 (−24.7 to −3.0)			−15.1 (−26.5 to −3.7)			−17.2 (−30.2 to −4.2)		
SP	0			0			0		
**TACL-4**	3.6 (1.1 – 6.0)	8.69	0.005^∗∗^	3.6 (1.1 – 6.2)	8.18	0.006^∗∗^	4.3 (1.4 – 7.2)	8.85	0.004^∗∗^

*POM-2, Pragmatics Observational Measure 2nd Edition; TACL-4, Test for Auditory Comprehension of Language 4th Edition; OT, Occupational Therapist; SP, Speech Pathologist; ^a^POM-2 scores from 3-month follow-up assessment in the clinic; ^∗^*p* < 0.05; ^∗∗^*p* < 0.01; ^∗∗∗^*p* < 0.001.*

**TABLE 6:** Results of multiple linear mixed model regression for SEE scores.

	**SEE Total**			**SEE Receptive**			**SEE Expressive**		
	**Parameter estimates (95%CI)**	***F***	***p***	**Parameter estimates (95%CI)**	***F***	***p***	**Parameter estimates (95%CI)**	***F***	***p***
**Time**		3.89	0.023^∗^		5.15	0.007^∗∗^		4.75	0.010^∗^
Pre	−0.22 (−6.5 to −3.1)			−0.26 (−0.50 to −0.01)			−0.24 (−0.50 to 0.01)		
Post	0.14 (−0.1 to 0.4)			0.14 (−0.11 to 0.38)			0.16 (−0.10 to 0.42)		
3-month follow-up	0			0			0		
**TACL-4**	0.12 (0.01 – 0.21)	4.89	0.031^∗^	0.08 (0.00 – 0.17)	4.08	0.048^∗^	–	–	–
**EVT-2**	0.03 (0.02 – 0.05)	16.64	< 0.001^∗∗∗^	0.04 (0.03 – 0.06)	32.31	< 0.001^∗∗∗^	0.03 (0.01 – 0.05)	15.82	< 0.001^∗∗∗^

*SEE = Social Emotional Evaluation; EM = estimated marginal; TACL-4 = Test for Auditory Comprehension of Language 4th Edition; EVT-2 = Expressive Vocabulary Test 2nd Edition; ^∗^*p* < 0.05; ^∗∗^*p* < 0.01; ^∗∗∗^*p* < 0.001.*

To understand the effect of therapist profession baseline TACL-4 and POM-2 scores of children seen by the occupational therapist were compared with those of children seen by the speech pathologist. No significant differences were present in baseline POM-2 scores, but TACL-4 scores were significantly lower for children seen by the occupational therapist, *t*(59) = −2.94, *p* = 0.05. However, as TACL-4 is also a significant variable within the multiple regression models, this difference does not explain the moderating effect of therapist profession. Conditional *R*^2^ was calculated to understand the variance in POM-2 scores explained by therapist profession using the method described by Nakagawa and Schielzeth (2013). Therapist profession accounted for 8.5, 8.8, and 6.7% of the variance in POM-2 Overall, Non-verbal and Verbal scores, respectively. This therapist comparison should be interpreted with caution, as only one therapist from each profession was involved.

## Discussion

The primary purpose of this randomized controlled trial was to evaluate the effectiveness of a play-based, peer-mediated intervention for improving pragmatic language in children with autism aged 6–11 years. Results indicated that the intervention is effective in improving non-verbal communication and overall pragmatic *performance* (POM-2) in children with autism during play-based interactions with a peer. The definition of pragmatic language adopted for this study includes verbal and non-verbal communication behaviors related to the emotional, social and communicative aspects of social language (Cordier et al., 2014). A previous systematic review of pragmatic language interventions for children with autism found that existing interventions targeted a limited range of these pragmatic language skills (Parsons et al., 2017), making this the first study to evaluate the effectiveness of an intervention for school-aged children with autism that targeted *all* aspects pragmatic language encompassed by contemporary definitions of the construct.

The use of a comprehensive observational measure of pragmatic language is also novel in the evaluation of pragmatic language interventions for school-aged children with autism. Prior to this study, a systematic review identified that children’s pragmatic language *performance* during a naturalistic social interaction had been evaluated as an outcome in only one pragmatic language intervention RCT for older children with autism (Parsons et al., 2017). However, the measure was narrow in focus, limited to capturing social initiations (Hopkins et al., 2011), and therefore provided little insight into performance of other pragmatic language skills. Results from the current study indicate it is possible for psychosocial interventions to have a positive impact on how children with autism enact pragmatic language skills during peer-peer play, suggesting a functional, performance focused approach to intervention and assessment is valid in this area.

Results from this study also demonstrated that changes in pragmatic language performance (POM-2) were maintained 3 months after the intervention period. Maintenance of treatment effects 3 months following a pragmatic language intervention has been evaluated following two previous RCTs for children with autism aged 6–11 years with mixed findings (Ryan and Charragain, 2010; Soorya et al., 2015). No RCT including children with autism aged 6–11 years has evaluated maintenance of treatment effects in pragmatics over a longer term (Parsons et al., 2017). There is a need for researchers to assess longer-term intervention effects to ensure benefits in targeted skills are maintained. Furthermore, investigations of longer-term benefits could also address friendship development, resilience, and self-worth.

Psychosocial interventions targeting pragmatic language do so with a broader aim of enhancing the daily social interactions of children, yet to date evaluations of intervention efficacy for school-aged children has not addressed whether targeted skills are enacted in ecologically valid social settings (Parsons et al., 2017). The current study was the first RCT to evaluate the range of pragmatic language skills applicable to school-aged children with autism during peer-to-peer social play interactions. Moreover, it was the first RCT to compare the pragmatic language performance of school-aged children with autism in multiple settings following an intervention. Results showed that children with autism demonstrated equivalent performance in the clinic and their homes at the end of the study, indicating maintenance and generalization of treatment effects to the home environment. Findings support the combined use of video-feedback, feedforward, peer-modeling, therapist-modeling, and parent mediation in conjunction with child-lead free-play to improve pragmatic language performance of children with autism, and that gains are maintained and generalized between clinic and home environments.

Interestingly, changes in children’s verbal pragmatic performance (POM-2 Verbal Communication Element) did not differ between children who did and did not receive the intervention, though verbal pragmatic performance did improve for all children over the intervention period, with maintenance 3 months later. Rasch analysis produces a person-item map to represent the spread of item difficulty within a measure. More difficult items sit at the top of the vertical axis, while easier items sit toward the bottom. Examination of the person-item map of all POM-2 items for this sample found that almost all of Verbal Communication Scale items appeared toward the top of the person-item map, indicating they represent the items on which the fewest participants performed at an “expert” level across the study (i.e., the most difficult items within the overall scale). As such, children may need more time to make greater gains in this area. Furthermore, therapists can place a consistent focus on verbal communication during the intervention period by (1) ensuring verbal communication skills are demonstrated and discussed in video-feedback on a weekly basis, and (2) facilitating the social play interactions where conversations are consistently maintained with both children making equal contributions.

Changes in pragmatic capacity (SEE) did not differ between children who did and did not receive the intervention. One reason for this may be the performance focus of the intervention components. For example, child-therapist discussions about pragmatic language during video-feedback concentrated on how skills can be enacted in contextualized practice, rather than explicit instruction to increase knowledge of unknown pragmatic rules. Practice effects might also explain the discord between results in pragmatic performance and capacity. Children in both groups could become more adept at responding to the items of the SEE as the time between tests was relatively short (i.e., 10-weeks). Conversely, even though the time interval was the same, children were unaware of the assessment criteria for the POM-2 and so practice effects are controlled for through the nature of the assessment. Another reason why pragmatic capacity changes were not different for the intervention-first and waitlist-first groups may be the way that SEE *z*-scores are calculated. The SEE’s authors report age-referenced *z*-scores are used for assessment interpretation. However, its subtests progress in difficulty, hence researchers have suggested that evaluation of subtest level competence may be diluted when subtests are conflated to derive composite scores (Elleseff, 2015).

A key finding of the moderator analysis was that the relationship between the children within dyads did not significantly predict the pragmatic language performance (POM-2) of children with autism. Parents have previously expressed a preference for inviting siblings as playmates due to concerns around placing burden on friends if they were asked to fill the role of playmate (Parsons et al., 2018). As siblings are the most frequently available playmate for children, and children with autism report having fewer quality friendships (Bauminger and Kasari, 2000), this finding contributes to both the feasibility and appropriateness of the intervention by supporting the use of siblings as playmates.

Children’s receptive syntax moderated pragmatic language performance and capacity scores in this study. Results reflect findings of previous meta-analyses showing that interventions for language content and form are most effective for children without concomitant receptive language difficulties (Law et al., 2004). This finding also reflects a body of evidence, which suggests a child’s ability to integrate spoken language with the social context for comprehension is associated with their structural language abilities (Norbury, 2005; Pijnacker et al., 2009). Care was taken within this study to present children with short, syntactically simple “catch phrases” to aid recall of targeted pragmatic language principles. Future development of the intervention might consider incorporating cues that are less linguistically laden (e.g., images, or gestures) to associate with the “catch phrases” and support comprehension for children with receptive language difficulties. Therapists must also ensure simple, concrete language is used during video-feedback discussions and within the playroom.

In this study, children’s pragmatic language performance scores (POM-2) were higher when the intervention was delivered by the speech pathologist than the occupational therapist, even when accounting for differences in receptive syntax scores. However, this result should be interpreted with caution and cannot be generalized as only one therapist from each profession was involved, this is the first time a speech pathologist has delivered this intervention, and therapist profession accounted for less than 10% of the variance in POM-2 scores. Implementing a play-based intervention for children with autism presents a prime opportunity for inter-professional collaboration between speech pathologists and occupational therapists. The model of play adopted for this intervention incorporated pragmatic language through the element of framing (Bundy, 2004); however, speech pathologists must consider all elements of the play model to ensure that the activities children engage in to practice targeted pragmatic language principles are in fact play. Similarly, the intervention provides occupational therapists with the opportunity to enhance children’s pragmatic language while targeting other elements of an important childhood occupation. Results suggest that future therapist training might consider providing occupational therapists with a more in-depth understanding of pragmatic language principles to maximize the integration of the play element of framing into clinical goal setting by both professions.

This study takes an important step toward addressing gaps in the pragmatic intervention literature by demonstrating maintenance and generalization of intervention effects. What is not yet known is whether effects generalize to social play interactions in other environments (i.e., school), with playmates who have not attended the intervention, or interactions with more than one peer. Future evaluation of children’s pragmatic language performance would establish the longer-term intervention effects, and consideration should be given to evaluating future friendship development and quality.

### Limitations

Although a majority of playmates were siblings who interacted on a regular basis, there is a possibility that children’s pragmatic language improved as a result of spending more time interacting with a playmate. This possible explanation could not be evaluated in this study due to the waitlisted control design. Future studies might consider an active control condition where non-sibling peers are also encouraged to interact regularly, but without any directed pragmatic language feedback or modeling.

One potential moderator that was not evaluated in this study is the pragmatic language abilities of the playmates. The playmates are an active ingredient in this intervention and it is reasonable to expect that their pragmatic abilities influenced the intervention effects for the children with autism. However, pragmatic language as measured by the POM-2 is a transaction between two individuals and as a result the scores of the playmates are dependent on the scores of the children with autism, and vice versa. In the context of this study, it is likely that the baseline POM-2 score of the playmates are an underestimation of their pragmatic language performance capabilities. Future studies might consider analyzing the POM-2 scores of the playmates to better understand the transactional nature of pragmatic language.

## Conclusion

We found that a peer-mediated, play-based intervention was effective in improving pragmatic language performance in children with autism aged 6–11 years. Gains were maintained in the short term and were observed in the home environment following the clinic-based intervention sessions. This intervention utilized a constellation of active treatment ingredients, including video-feedback, video-feedforward, peer- and therapist-modeling, and parent mediation within the context of child-lead free-play to improve pragmatic language performance of children with autism. As yet, we do not know which intervention ingredients are specifically driving these intervention effects – we leave this for future investigation. Further research is also required to understand generalization of skills to other social contexts (e.g., school), how best to support change for children with concurrent structural language difficulties, and appropriate training methods for therapists.

## Data Availability

The datasets generated for this study are available on request to the corresponding author.

## Ethics Statement

This study was carried out in accordance with the recommendations of the Curtin University Human Research Ethics Committee, with written informed consent from all subjects. All subjects gave written informed consent in accordance with the Declaration of Helsinki. The protocol was approved by the Curtin University Human Research Ethics Committee.

## Author Contributions

All authors conceived and designed the study, revised the manuscript, and read and approved the submitted version. LP collected the data and performed the statistical analysis under the supervision of RC, NM, and AJ. LP wrote the first draft of the manuscript.

## Conflict of Interest Statement

The authors declare that the research was conducted in the absence of any commercial or financial relationships that could be construed as a potential conflict of interest.

## References

[B1] Adams C. Baxendale B. Lloyd J. Aldred C. (2005). Pragmatic language impairment: case studies of social and pragmatic language therapy. *Child Lang. Teach. Ther.* 21 227–250. 10.1191/0265659005ct290oa

[B2] Adams C. Lockton E. Freed J. Gaile J. Earl G. McBean K. (2012). The social communication intervention project: a randomized controlled trial of the effectiveness of speech and language therapy for school-age children who have pragmatic and social communication problems with or without autism spectrum disorder. *Int. J. Lang. Commun. Disord.* 47 233–244. 10.1111/j.1460-6984.2011.00146.x 22512510

[B3] Adobe Systems Incorporated (2014). *Adobe Premier Pro CC (Version 8.1.0 (81) Build).* San Jose, CA: Adobe Systems Incorporated.

[B4] American Psychiatric Association (2000). *Diagnostic and Statistical Manual of Mental Disorders*, 4th Edn Washington, DC: American Psychiatric Association.

[B5] American Psychiatric Association (2013). *Diagnostic and Statistical Manual of Mental Disorders*, 5th Edn Washington, DC: American Psychiatric Association.

[B6] Bauminger N. Kasari C. (2000). Loneliness and friendship in high-functioning children with autism. *Child Dev.* 71 447–456. 10.5353/th_b4171656 10834476

[B7] Bauminger N. Solomon M. Aviezer A. Heung K. Gazit L. Brown J. (2008). Children with autism and their friends: a multidimensional study of friendship in high-functioning autism spectrum disorder. *J. Abnorm. Child Psychol.* 36 135–150. 10.1007/s10802-007-9156-x 18172754

[B8] Beaumont R. Sofronoff K. (2008). A multi-component social skills intervention for children with Asperger syndrome: the junior detective training program. *J. Child Psychol. Psychiatry* 49 743–753. 10.1111/j.1469-7610.2008.01920.x 18503531

[B9] Begeer S. Koot H. M. Rieffe C. Terwogt M. M. Stegge H. (2008). Emotional competence in children with autism: diagnostic criteria and empirical evidence. *Dev. Rev.* 28 342–369. 10.1016/j.dr.2007.09.001

[B10] Bellini S. Akullian J. (2007). A meta-analysis of video modeling and video self-modeling interventions for children and adolescents with autism spectrum disorders. *Except. Child.* 73 264–287. 10.1177/001440290707300301

[B11] Bishop D. (2006). *Children’s Communication Checklist*, 2nd Edn San Antonio, TX: Pearson.

[B12] Bundy A. (2004). *Test of Playfulness*, 4th Edn Sydney, NSW: The University of Sydney.

[B13] Carrow-Woolfolk E. (2014). *Test for Auditory Comprehension of Language*, 4th Edn Greenville, SC: Super Duper Publications.

[B14] Chang Y.-C. Locke J. (2016). A systematic review of peer-mediated interventions for children with autism spectrum disorder. *Res. Autism Spectr. Disord.* 27 1–10. 10.1016/j.rasd.2016.03.010 27807466 PMC5087797

[B15] Cohen J. (1988). *Statistical Power Analysis for the Behavioral Sciences*, 2nd Edn Hillsdale, NJ: Earlbaum.

[B16] Conners C. K. (2008). *Conners Comprehensive Behavior Rating Scales*, 3rd Edn Toronto, ON: Multi-Health Systems Inc.

[B17] Corbett B. A. Key A. P. Qualls L. Fecteau S. Newsom C. Coke C. (2015). Improvement in social competence using a randomized trial of a theatre intervention for children with autism spectrum disorder. *J. Autism Dev. Disord.* 46 658–672. 10.1007/s10803-015-2600-9 26419766 PMC5633031

[B18] Cordier R. Bundy A. Hocking C. Einfeld S. (2009). A model for play-based intervention for children with ADHD. *Austr. Occupat. Ther. J.* 56 332–340. 10.1111/j.1440-1630.2009.00796.x 20854539

[B19] Cordier R. Munro N. Wilkes-Gillan S. Speyer R. Parsons L. Joosten A. (2019). Applying item response theory (IRT) modelling to an observational measure of childhood pragmatics: the pragmatics observational measure-2. *Front. Psychol.* 10:408. 10.3389/fpsyg.2019.00408 30873094 PMC6403159

[B20] Cordier R. Munro N. Wilkes-Gillan S. Speyer R. Pearce W. M. (2014). Reliability and validity of the pragmatics observational measure (POM): a new observational measure of pragmatic language for children. *Res. Dev. Disabil.* 35 1588–1598. 10.1016/j.ridd.2014.03.050 24769431

[B21] Craig P. Dieppe P. Macintyre S. Michie S. Nazareth I. Petticrew M. (2008). Developing and evaluating complex interventions: the new medical research council guidance. *BMJ* 337 979–983. 10.1136/bmj.a1655 18824488 PMC2769032

[B22] DeRosier M. E. Swick D. C. Davis N. O. McMillen J. S. Matthews R. (2011). The efficacy of a social skills group intervention for improving social behaviors in children with high functioning autism spectrum disorders. *J. Autism Dev. Disord.* 41 1033–1043. 10.1007/s10803-010-1128-2 21042870

[B23] Elleseff T. (2015). Assessing social communication abilities of school-aged children. *Perspect. School Based Issues* 16 79–86. 10.1044/sbi16.3.79

[B24] Faul F. Erdfelder E. Lang A.-G. Buchner A. (2007). G^∗^ Power 3: a flexible statistical power analysis program for the social, behavioral, and biomedical sciences. *Behav. Res. Methods* 39 175–191. 10.3758/bf03193146 17695343

[B25] Fujiki M. Brinton B. Clarke D. (2002). Emotion regulation in children with specific language impairment. *Lang. Speech Hear. Serv. Schools* 33 102–111. 10.1044/0161-1461(2002/008) 27764463

[B26] Gifford-Smith M. E. Brownell C. A. (2003). Childhood peer relationships: social acceptance, friendships, and peer networks. *J. School Psychol.* 41 235–284. 10.1016/s0022-4405(03)00048-7

[B27] Glasson E. J. MacDermott S. Dixon G. Cook H. Chauvel P. Maley-Berg A. (2008). Management of assessments and diagnoses for children with autism spectrum disorders: the Western Australian model. *Med. J. Austr.* 188 288–291. 10.5694/j.1326-5377.2008.tb01623.x 18312193

[B28] Haahr M. (2010). *Random.org**: True Random Number Service.* Available at: http://www.random.org (accessed February 8, 2016).

[B29] Henning B. Cordier R. Wilkes-Gillan S. Falkmer T. (2016). A pilot play-based intervention to improve the social play interactions of children with autism spectrum disorder and their typically developing playmates. *Austr. Occupat. Ther. J.* 63 223–232. 10.1111/1440-1630.12285 27118688

[B30] Hoffmann T. C. Glasziou P. P. Boutron I. Milne R. Perera R. Moher D. (2014). Better reporting of interventions: template for intervention description and replication (TIDieR) checklist and guide. *BMJ* 348:g1687. 10.1136/bmj.g1687 24609605

[B31] Hopkins I. M. Gower M. W. Perez T. A. Smith D. S. Amthor F. R. Wimsatt F. (2011). Avatar assistant: improving social skills in students with an ASD through a computer-based intervention. *J. Autism Dev. Disord.* 41 1543–1555. 10.1007/s10803-011-1179-z 21287255

[B32] IBM Corporation (2013). *IBM SPSS Statistics (Version 22).* Armonk, NY: IBM Corp.

[B33] Kent C. Cordier R. Joosten A. Wilkes-Gillan S. Bundy A. (2018). Peer-mediated intervention to improve play skills in children with autism spectrum disorder: a feasibility study. *Austr. Occupat. Ther. J.* 65 176–186. 10.1111/1440-1630.12459 29527682

[B34] Law J. Garrett Z. Nye C. (2004). The efficacy of treatment for children with developmental speech and language delay/disorder: a meta-analysis. *J. Speech Lang. Hear. Res.* 47 924–943. 10.1044/1092-4388(2004/069) 15324296

[B35] Linacre J. (2016). *WINSTEPS Rasch Measurement Computer Program (Version 3.92*.0). Chicago, IL: WINSTEPS.com.

[B36] Lockton E. Adams C. Collins A. (2016). Do children with social communication disorder have explicit knowledge of pragmatic rules they break? A comparison of conversational pragmatic ability and metapragmatic awareness. *Int. J. Lang. Commun. Disord.* 51 508–517. 10.1111/1460-6984.12227 26916221

[B37] Lopata C. Thomeer M. L. Rodgers J. D. Donnelly J. P. McDonald C. A. (2016). RCT of Mind Reading as a component of a psychosocial treatment for high-functioning children with ASD. *Res. Autism Spectr. Disord.* 21 25–36. 10.1016/j.rasd.2015.09.003

[B38] Lopata C. Thomeer M. L. Volker M. A. Toomey J. A. Nida R. E. Lee G. K. (2010). RCT of a manualized social treatment for high-functioning autism spectrum disorders. *J. Autism Dev. Disord.* 40 1297–1310. 10.1007/s10803-010-0989-8 20232240

[B39] Matthews D. Biney H. Abbot-Smith K. (2018). Individual differences in children’s pragmatic ability: a review of associations with formal language, social cognition, and executive functions. *Lang. Learn. Dev.* 14 186–223. 10.1080/15475441.2018.1455584

[B40] Mundy P. Sigman M. Ungerer J. Sherman T. (1986). Defining the social deficits of autism: the contribution of non-verbal communication measures. *J. Child Psychol. Psychiatry* 27 657–669. 10.1111/j.1469-7610.1986.tb00190.x 3771682

[B41] Nakagawa S. Schielzeth H. (2013). A general and simple method for obtaining R2 from generalized linear mixed-effects models. *Methods Ecol. Evol.* 4 133–142. 10.1111/j.2041-210x.2012.00261.x 30239975

[B42] Norbury C. F. (2005). Barking up the wrong tree? Lexical ambiguity resolution in children with language impairments and autistic spectrum disorders. *J. Exp. Child Psychol.* 90 142–171. 10.1016/j.jecp.2004.11.003 15683860

[B43] Parsons L. Cordier R. Munro N. Joosten A. (2018). The feasibility and appropriateness of a peer-to-peer, play-based intervention for improving pragmatic language in children with autism spectrum disorder. *Int. J. Speech Lang. Pathol.* 2 1–13. 10.1080/17549507.2018.1492630 30175619

[B44] Parsons L. Cordier R. Munro N. Joosten A. Speyer R. (2017). A systematic review of pragmatic language interventions for children with autism spectrum disorder. *PLoS One* 12:e0172242. 10.1371/journal.pone.0172242 28426832 PMC5398499

[B45] Paul R. Orlovski S. M. Marcinko H. C. Volkmar F. (2009). Conversational behaviors in youth with high-functioning ASD and Asperger syndrome. *J. Autism Dev. Disord.* 39 115–125. 10.1007/s10803-008-0607-1 18607708 PMC2819316

[B46] Pijnacker J. Hagoort P. Buitelaar J. Teunisse J.-P. Geurts B. (2009). Pragmatic inferences in high-functioning adults with autism and Asperger syndrome. *J. Autism Dev. Disord.* 39 607–618. 10.1007/s10803-008-0661-8 19052858

[B47] Prutting C. A. Kirchner D. M. (1987). A clinical apraisal of the pragmatic aspects of language. *J. Speech Hear. Disord.* 52 105–119. 10.1044/jshd.5202.105 3573742

[B48] Rodas N. V. Eisenhower A. Blacher J. (2017). Structural and pragmatic language in children with ASD: longitudinal impact on anxiety and externalizing behaviors. *J. Autism Dev. Disord.* 47 3479–3488. 10.1007/s10803-017-3265-3 28776120

[B49] Ryan C. Charragain C. N. (2010). Teaching emotion recognition skills to children with autism. *J. Autism Dev. Disord.* 40 1505–1511. 10.1007/s10803-010-1009-8 20386975

[B50] Schulz K. F. Altman D. G. Moher D. (2010). CONSORT 2010 statement: updated guidelines for reporting parallel group randomised trials. *BMC Med.* 8:32. 10.1186/1745-6215-11-32 20334633 PMC2860339

[B51] Semel E. M. Wiig E. Secord W. (2003). *Clinical Evaluation of Language Fundamentals*, 4th Edn San Antonio, TX: Psychological Corporation.

[B52] Soorya L. V. Siper P. M. Beck T. Soffes S. Halpern D. Gorenstein M. (2015). Randomized comparative trial of a social cognitive skills group for children with autism spectrum disorder. *J. Am. Acad. Child Adolesc. Psychiatry* 54 208–216. 10.1016/j.jaac.2014.12.005 25721186 PMC4346205

[B53] St Clair M. C. Pickles A. Durkin K. Conti-Ramsden G. (2011). A longitudinal study of behavioral, emotional and social difficulties in individuals with a history of specific language impairment (SLI). *J. Commun. Disord.* 44 186–199. 10.1016/j.jcomdis.2010.09.004 20970811

[B54] Thomeer M. L. Smith R. A. Lopata C. Volker M. A. Lipinski A. M. Rodgers J. D. (2015). Randomized controlled trial of Mind Reading and in vivo rehearsal for high-functioning children with ASD. *J. Autism Dev. Disord.* 45 2115–2127. 10.1007/s10803-015-2374-0 25643864

[B55] Tobin M. C. Drager K. D. Richardson L. F. (2014). A systematic review of social participation for adults with autism spectrum disorders: Support, social functioning, and quality of life. *Res. Autism Spectr. Disord.* 8 214–229. 10.1016/j.rasd.2013.12.002

[B56] Watkins L. O’Reilly M. Kuhn M. Gevarter C. Lancioni G. E. Sigafoos J. (2015). A review of peer-mediated social interaction interventions for students with autism in inclusive settings. *J. Autism Dev. Disord.* 45 1070–1083. 10.1007/s10803-014-2264-x 25272953

[B57] Westby C. Washington K. N. (2017). Using the international classification of functioning, disability and health in assessment and intervention of school-aged children with language impairments. *Lang. Speech Hear. Serv. Schools* 48 137–152. 10.1044/2017_lshss-16-0037 28630972

[B58] Wiig E. (2008). *Social Emotional Evaluation.* Greenville, CA: Super Duper Publications.

[B59] Wilkes-Gillan S. Bundy A. Cordier R. Lincoln M. Chen Y.-W. (2016). A randomised controlled trial of a play-based intervention to improve the social play skills of children with attention deficit hyperactivity disorder (ADHD). *PLoS One* 11:e0160558. 10.1371/journal.pone.0160558 27529693 PMC4987013

[B60] Williams K. T. (2007). *Expressive Vocabulary Test*, 2nd Edn Bloomington, MN: Pearson.

[B61] World Health Organization (2001). *International Classification of Functioning, Disability and Health: ICF.* Geneva: World Health Organization.

