# Supplementary material for: A Randomized Controlled Trial of a Play-Based, Peer-Mediated Pragmatic Language Intervention for Children With Autism
Source: Front Psychol. 2019 Aug 27;10:1960. doi: 10.3389/fpsyg.2019.01960 (PMC6776827; doi:10.3389/fpsyg.2019.01960)
Supplement: Supplementary file 1 [file Data_Sheet_1.PDF]

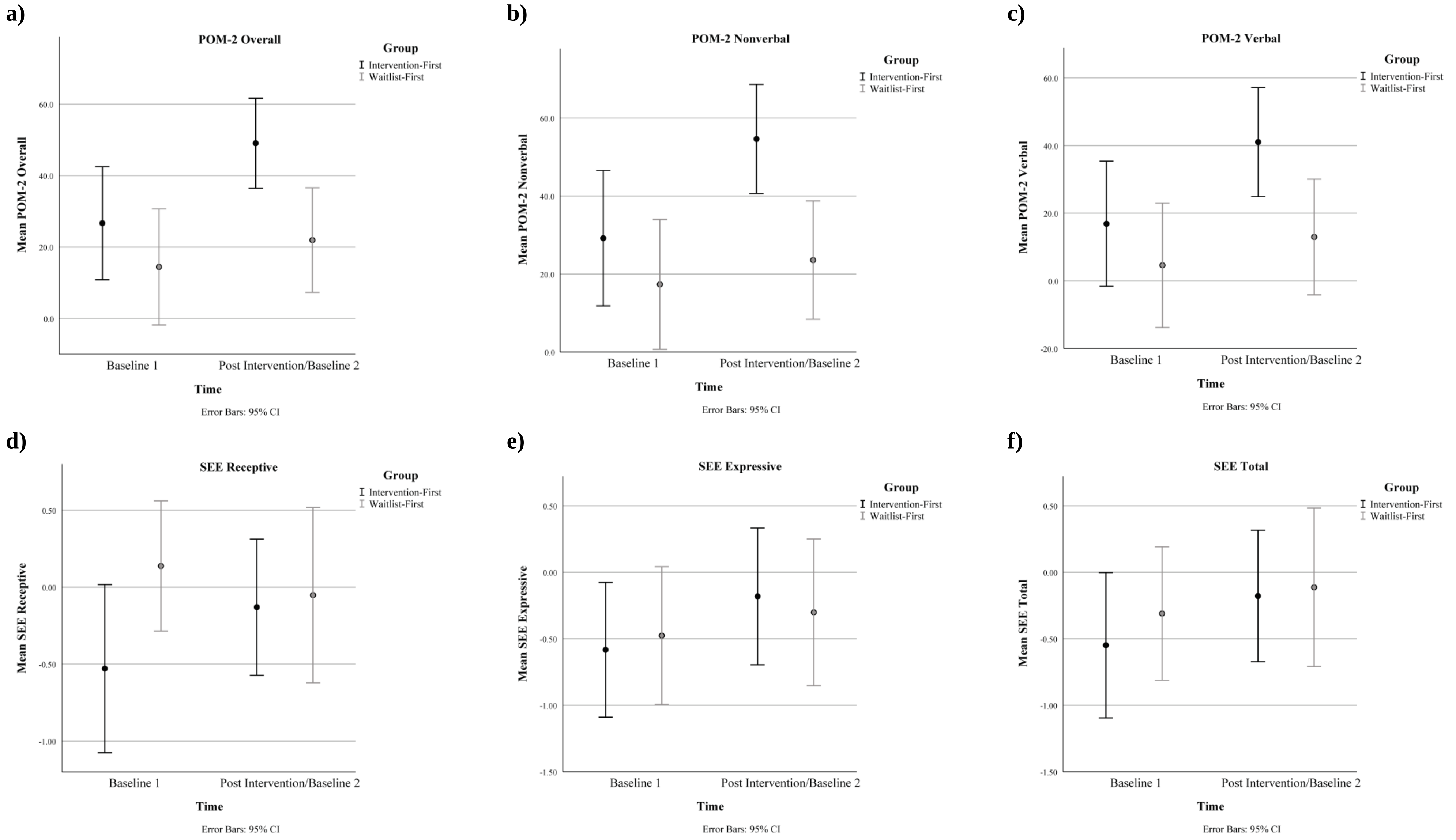

Supplementary Figure 1. Pre- and post- scores for intervention-first group and waitlist-first group on all outcome measures.
Note: POM-2 = Pragmatics Observational Measure 2nd Edition; SEE = Social-Emotional Evaluation
